# Supplementary material for: External validation of a decision rule for bacteremia vs contaminants in pediatric blood cultures
Source: PLoS One. 2026 Nov 13;21(11):e0355010. doi: 10.1371/journal.pone.0355010 (PMC13766231; doi:10.1371/journal.pone.0355010)
Supplement: Supplementary file 1 [file pone.0355010.s005.pdf]

PLOS One

External validation of a decision rule for bacteremia vs contaminants in pediatric blood cultures

--Manuscript Draft--

**Manuscript Number:**	PONE-D-26-35518
**Article Type:**	Research Article
**Full Title:**	External validation of a decision rule for bacteremia vs contaminants in pediatric blood cultures
**Short Title:**	External validation of a pediatric bacteremia rule
**Corresponding Author:**	Johan N Siebert, MD, PD<br>Geneva University Hospitals Children's Hospital: Hopitaux Universitaires Geneve Hopital des Enfants<br>Geneva, SWITZERLAND
**Keywords:**	Bacteremia; Blood Culture; Clinical decision rules; Pediatrics; Emergency Service, Hospital
**Abstract:**	BACKGROUND: Half of positive blood cultures in pediatric emergency departments (PEDs) represent contaminants, driving unnecessary hospitalization, antibiotic exposure, and repeat visits. A clinical decision rule derived at CHU Sainte-Justine showed 99% sensitivity and 60% specificity for distinguishing bacteremia from contaminants but had not been externally validated. We sought to validate this rule in an independent pediatric cohort.<br>METHODS: This retrospective diagnostic study spanned from January 2015 to May 2025 at a tertiary PED in Switzerland, using positive blood cultures from patients younger than 16 years. The four predictors (Gram-negative organisms or Gram-positive cocci in pairs or chains; time to positivity <17 hours; indwelling device; suspected osteoarticular infection) classified each case as low, moderate, or high risk. The primary outcome was bacteremia, adjudicated by two independent reviewers, based on organism identity and infectious disease specialist's assessment. Diagnostic accuracy was assessed with 95% CIs.<br>RESULTS: Of 130 children enrolled (median age 3.8 years [IQR 0.9-9.9]; 61.5% male), 78 (60.0%) had true bacteremia. The rule yielded a sensitivity of 97.4% (95% CI, 91.0-99.7), specificity of 69.2% (95% CI, 54.9-81.3), positive predictive value of 82.6% (95% CI, 73.3-89.7), and negative predictive value of 94.7% (95% CI, 82.3-99.4). Both false-negatives were immunocompetent children with methicillin-susceptible Staphylococcus aureus bacteremia without indwelling devices. Among contaminants, 71% received antibiotics under usual care versus 31% classified as moderate or high risk by the rule.<br>CONCLUSIONS: This first external validation supports the Sainte-Justine rule in a distinct pediatric population, preserving sensitivity with higher specificity. Multicenter validation is warranted before adoption.
**Order of Authors:**	Madeleine D’Amours-Gravel, MS
	Alessia Charvet, MS
	Carla Ibañez Miguel, MS
	Nine Rouxel, MS
	Camille Fontaine, MS
	Julie Besson, MS
	Léna Jiguet, MS
	Liam Karara, MS
	Luna Pozzi, MS
	Claudia Teixeira, MS
	Carla Henoud-Bertaina, MS

Powered by Editorial Manager® and ProduXion Manager® from Aries Systems Corporation

	Céline Alves, MS
	Abdessalam Cherkaoui, PhD, FAMH
	Delphine S. Courvoisier, PhD, Prof.
	Johan N Siebert, MD, PD, CC
**Additional Information:**	
**Question**	**Response**
**Financial Disclosure**<br><br>Enter a financial disclosure statement that describes the sources of funding for the work included in this submission. Review the submission guidelines for detailed requirements. View published research articles from *PLOS ONE* for specific examples.<br><br>This statement is required for submission and **will appear in the published article** if the submission is accepted. Please make sure it is accurate.<br><br>**Funded studies**<br>Enter a statement with the following details:<br>• Initials of the authors who received each award<br>• Grant numbers awarded to each author<br>• The full name of each funder<br>• URL of each funder website<br>• Did the sponsors or funders play any role in the study design, data collection and analysis, decision to publish, or preparation of the manuscript?<br><br>Did you receive funding for this work?	The author(s) received no specific funding for this work.
**Competing Interests**<br><br>Use the instructions below to enter a competing interest statement for this submission. On behalf of all authors, disclose any competing interests that could be perceived to bias this work—acknowledging all financial support and any other relevant financial or non-financial competing interests.	The authors have declared that no competing interests exist.

This statement is required for submission and **will appear in the published article** if the submission is accepted. Please make sure it is accurate and that any funding sources listed in your Funding Information later in the submission form are also declared in your Financial Disclosure statement.<br><br>View published research articles from *PLOS ONE* for specific examples.<br><br>**NO authors have competing interests**<br><br>Enter: *The authors have declared that no competing interests exist.*<br><br>**Authors with competing interests**<br><br>Enter competing interest details beginning with this statement:<br><br>*I have read the journal's policy and the authors of this manuscript have the following competing interests: [insert competing interests here]*<br><br>* typeset	
**Ethics Statement**<br><br>Enter an ethics statement for this submission. This statement is required if the study involved:<br><br>• Human participants<br>• Human specimens or tissue<br>• Vertebrate animals or cephalopods<br>• Vertebrate embryos or tissues<br>• Field research<br><br>Write "N/A" if the submission does not require an ethics statement.<br><br>General guidance is provided below. Consult the submission guidelines for detailed instructions. **Make sure that all information entered here is included in the Methods section of the manuscript.**	Ethical approval was obtained from the Geneva Research Ethics Commission (CCER, approval number: 2025–01069) and written informed consent was obtained for all research individuals.

**Format for specific study types**<br><br>**Human Subject Research (involving human participants and/or tissue)**<br>• Give the name of the institutional review board or ethics committee that approved the study<br>• Include the approval number and/or a statement indicating approval of this research<br>• Indicate the form of consent obtained (written/oral) or the reason that consent was not obtained (e.g. the data were analyzed anonymously)<br><br>**Animal Research (involving vertebrate animals, embryos or tissues)**<br>• Provide the name of the Institutional Animal Care and Use Committee (IACUC) or other relevant ethics board that reviewed the study protocol, and indicate whether they approved this research or granted a formal waiver of ethical approval<br>• Include an approval number if one was obtained<br>• If the study involved *non-human primates*, add *additional details* about animal welfare and steps taken to ameliorate suffering<br>• If anesthesia, euthanasia, or any kind of animal sacrifice is part of the study, include briefly which substances and/or methods were applied<br><br>**Field Research**<br><br>Include the following details if this study involves the collection of plant, animal, or other materials from a natural setting:<br>• Field permit number<br>• Name of the institution or relevant body that granted permission	
**Data Availability**<br><br>Before publication, Authors are required to make fully available and without restriction all data underlying their findings. Sharing the data underlying your findings increases the visibility, credibility, and impact of your work, while supporting transparency and future scientific	The data consist of individual patient records extracted from the electronic health record of Geneva University Hospitals, Switzerland. Individual-level data cannot be shared publicly because they are sensitive patient data, and public release is precluded by the terms of the ethics approval and patient consent and by the risk of reidentification in a single-center pediatric cohort. The datasets generated during and/or analysed during the current study are available in deidentified form from the corresponding author on reasonable request.

Powered by Editorial Manager® and ProduXion Manager® from Aries Systems Corporation

discovery. Please see our PLOS Data Policy page for detailed information on this policy.<br><br>A **Data Availability Statement**, detailing where the data can be accessed, is required at first submission. Insert your Data Availability Statement in the box below.<br><br>Please see the data reporting section of our submission guidelines for instructions on what you need to include in your Data Availability Statement. Examples of acceptable Data Availability Statements are included here for reference:<br>1. The minimal data set [and accompanying code, where generated] is available at [repository name] via [DOI hyperlink]<br>2. No data was generated by this study. The following existing data sources were used: [dataset name] from [dataset location] available via [web address or DOI hyperlink].<br>3. For Study Protocols: No datasets were generated or analysed during the current study. All relevant data from this study will be made available upon study completion.<br><br>*PLOS allows rare exemptions to address legal and ethical concerns. If you have legal or ethical restrictions, please use the box below to detail these in full sentences for the Journal team to consider.*<br><br>This statement will be typeset if the manuscript is accepted for publication.	

Manuscript
Click here to access/download;Manuscript;Core manuscript_PLOSOne_RV1.docx

External validation of a decision rule for bacteremia vs contaminants in pediatric blood cultures

Short title: External validation of a pediatric bacteremia rule

Madeleine D'amours-Gravel[1], Alessia Charvet[2], Carla Ibañez Miguel[2], Nine Rouxel[2], Camille Fontaine[2], Julie Besson[2], Léna Jiguet[2], Liam Karara[2], Luna Pozzi[2], Claudia Teixeira[2], Carla Henoud-Bertaina[2], Céline Alves[2], Abdessalam Cherkaoui[3], Delphine S. Courvoisier[4], Johan N. Siebert[2,5*]

[1] Faculty of Medicine, University of Montreal, Montreal, Québec, Canada

[2] Faculty of Medicine, University of Geneva, Geneva, Switzerland

[3] Department of Diagnostics, Division of Laboratory Medicine, Bacteriology Laboratory, Geneva University Hospitals, Geneva, Switzerland

[4] Quality of Care Division, Medical directorate, Geneva University Hospitals, Geneva, Switzerland

[5] Department of Pediatric Emergency Medicine, Geneva Children's Hospital, Geneva University Hospitals, Geneva, Switzerland

*** Corresponding author:**
E-mail: Johan.Siebert@hug.ch (JNS)

Abstract

BACKGROUND: Half of positive blood cultures in pediatric emergency departments (PEDs) represent contaminants, driving unnecessary hospitalization, antibiotic exposure, and repeat visits. A clinical decision rule derived at CHU Sainte-Justine showed 99% sensitivity and 60% specificity for distinguishing bacteremia from contaminants but had not been externally validated. We sought to validate this rule in an independent pediatric cohort.

METHODS: This retrospective diagnostic study spanned from January 2015 to May 2025 at a tertiary PED in Switzerland, using positive blood cultures from patients younger than 16 years. The four predictors (Gram-negative organisms or Gram-positive cocci in pairs or chains; time to positivity <17 hours; indwelling device; suspected osteoarticular infection) classified each case as low, moderate, or high risk. The primary outcome was bacteremia, adjudicated by two independent reviewers, based on organism identity and infectious disease specialist's assessment. Diagnostic accuracy was assessed with 95% CIs.

RESULTS: Of 130 children enrolled (median age 3.8 years [IQR 0.9-9.9]; 61.5% male), 78 (60.0%) had true bacteremia. The rule yielded a sensitivity of 97.4% (95% CI, 91.0-99.7), specificity of 69.2% (95% CI, 54.9-81.3), positive predictive value of 82.6% (95% CI, 73.3-89.7), and negative predictive value of 94.7% (95% CI, 82.3-99.4). Both false-negatives were immunocompetent children with methicillin-susceptible *Staphylococcus aureus* bacteremia without indwelling devices. Among contaminants, 71% received antibiotics under usual care versus 31% classified as moderate or high risk by the rule.

CONCLUSIONS: This first external validation supports the Sainte-Justine rule in a distinct pediatric population, preserving sensitivity with higher specificity. Multicenter validation is warranted before adoption.

1. Introduction

1.1 Background

Fever accounts for 10 to 20% of pediatric emergency department (PED) visits and is the most common chief complaint among infants and children [1, 2]. Bacteremia is the underlying cause in 1.5% to 2.3% of these patients [3]. Blood cultures are routinely obtained in febrile children, where guidelines strongly support their use [4, 5], and in those with suspected invasive bacterial infection [6, 7]. Although timely recognition of true bacteremia is critical given the risk of progression to meningitis, septic shock, metastatic infections, or death [8-10], 40% to 60% of positive blood cultures represent contaminants [11, 12]. This figure reflects a low underlying prevalence of true bacteremia rather than high absolute contamination rates, which remain in the 1.5 to 6.5% range across all PED blood cultures [13, 14].

1.2 Importance

False-positive results carry measurable clinical and economic consequences [15]. Children with contaminated cultures often return to the PED, undergo additional venipunctures, are admitted unnecessarily, and receive empirical antibiotics, with attendant risks of adverse events, line-related complications, and resistance selection [4, 12, 13, 16, 17]. A multicenter survey confirmed substantial management heterogeneity. In identical scenarios involving a positive blood culture, 49% of respondents would have discharged the patient whereas 35% would have admitted for intravenous antibiotics [18]. This variability persists despite American Academy of Pediatrics guidance [4] and the PECARN febrile-infant rule [19], neither of which addresses the management of a positive blood culture once obtained.

Real-time molecular diagnostics, including multiplex polymerase chain reaction (PCR), syndromic panels, and metagenomic next-generation sequencing are increasingly used alongside conventional blood cultures and can accelerate pathogen identification and antimicrobial optimization. Their contribution to the specific task of separating true bacteremia from contamination is, however, limited. Their targeted panels miss organisms not included in the assay, with recognized coverage gaps in pediatric populations [20]. Their high analytical sensitivity detects nucleic acid from both viable and non-viable organisms, complicating discrimination of true pathogens [21]. And their results require careful correlation with the clinical presentation and other microbiological findings [22]. High cost of instruments, reagents, and maintenance further limits widespread implementation [23].

Several predictors discriminate true bacteremia from contaminants, including Gram-stain morphology, time to positivity (TTP), inflammatory markers, indwelling device, and suspected osteoarticular infection [11, 24-27]. TTP appears particularly informative, with shorter values for true bacteremia and 90% of case become positive within 24 hours [25]. Procalcitonin and C-reactive protein, although useful for invasive bacterial infections in young febrile infants [28, 29], are of limited value for this specific distinction. Combining such predictors into a standardized decision tool is therefore the logical next step. The Hospital for Sick Children algorithm, externally validated in Montreal, achieved 100% sensitivity but only 11% specificity [11], an imbalance that limits clinical utility. More recently, Gravel et al. derived and internally validated a four-predictor clinical decision

rule (CDR) in children with positive blood cultures, yielding 99% sensitivity and 60% specificity [24].

1.3 Goals of this investigation

Because clinical prediction models require external validation in independent settings before safe and generalizable adoption [30, 31], and none has been published for this rule, our objective was to perform the first external validation of the Sainte-Justine four-predictor CDR in an independent pediatric cohort.

2. Methods

2.1 Study design and setting

We conducted a retrospective, single-center cohort study from January 1, 2015 to May 31, 2025 in the tertiary PED of the Geneva University Hospitals (HUG), Switzerland, managing approximately 36,000 visits per year. The bacteriology laboratory processes blood cultures using continuous-monitoring automated incubation BD BACTEC™FX systems that flag positivity 24 hours per day. In 2024, 78,768 blood culture bottles were submitted (positivity rate 6.2%) [32], with delayed loading over 24 hours in 0.9%. Laboratory operations run from 7:30 A.M. daily, closing at 7:30 P.M. on weekdays, 5:00 P.M. on Saturdays, and 1:00 P.M. on Sundays. Ethical approval was obtained from the Geneva Research Ethics Commission (CCER, approval number: 2025–01069) and written informed consent was obtained for all research individuals. The study was in accordance with the Helsinki Declaration [33] and followed applicable reporting guidelines [30, 34, 35].

2.2 Participants

Inclusion criteria. Children younger than 16 years at the PED visit, with at least one positive blood culture drawn during that visit, and written informed consent for secondary use of medical data from a legal representative or the participant.

Exclusion criteria. Age 16 years or older at the index visit; negative or inconclusive blood cultures; absence of a recorded TTP value or other incomplete records for variables required to apply the CDR; and ambiguous diagnostic information unresolved by independent adjudication.

Eligible cases were identified through the institutional microbiology database and cross-referenced against the electronic health record (EHR). To minimize selection bias, they were randomly ordered before screening and enrollment continued until the prespecified sample size was achieved. Because recruitment was capped at the target sample size, positive cultures not screened by that point were not assessed. Enrolled patients therefore represented a random subset of all eligible cases rather than a clinically selected sample.

2.3 Outcomes

The primary outcome was true bacteremia, defined with the same two-step adjudication approach used in the derivation study [24], enabling direct comparability between cohorts. The reference-standard was established independently and prior to the CDR result. For each positive culture, two independent reviewers (MDG, JNS) classified the result as true bacteremia or contaminant on the basis of 1) the identity of the organism, categorized using

the Centers for Disease Control and Prevention list of common commensals [36] and the reference categorization of probable contaminants [37], and 2) the pediatric infectious disease specialist's report documented in the EHR. Disagreements were resolved by consensus, with a third reviewer available for arbitration. The four CDR predictors were extracted and the risk classification was computed only afterward. C-reactive protein and procalcitonin were recorded as descriptive variables only and contributed neither to the reference-standard adjudication nor to the CDR classification.

2.4 Predictors

The four CDR predictors were derived by the original authors from a literature review and expert consensus, then selected through Classification and Regression Tree modeling optimized for sensitivity. We applied them in the sequential structure (Fig 1) proposed by Gravel et al. [24]:

- Gram-negative organisms OR Gram-positive cocci arranged in pairs or chains on initial Gram stain (high-risk gate).
- TTP below 17 hours from placement of the bottle into the BD BACTEC™FX system to detection of microbial growth.
- Indwelling devices (S1 Table).
- Clinical suspicion of osteoarticular infection (septic arthritis or osteomyelitis at the index visit).

Fig 1. The Sainte-Justine Clinical Decision Rule for Pediatric Positive Blood Cultures. Sequential application of the four predictors classifies patients into three risk strata. Patients meeting the Gram-stain criterion (Gram-negative bacteria or Gram-positive organisms in pairs or chains) are classified as high risk. Among the remainder, those with at least one of the three

secondary criteria (indwelling device, suspicion of osteoarticular infection, or TTP < 17 hours) are classified as moderate risk. Patients meeting none of the four criteria are classified as low risk of true bacteremia. Adapted with permission from Gravel et al [24].

2.5 Data Extraction

Data were first accessed on July 9, 2025. Each chart was independently reviewed by two investigators (MDG, JNS) using a structured case report form derived from the original Sainte-Justine instrument. Extracted variables included demographics, comorbidities, presenting features, laboratory and microbiological variables, and management data (S2 Table).

2.6 Sample Size

Based on a true bacteremia prevalence of approximately 50% in positive pediatric blood cultures in the derivation study [24], we estimated that 130 evaluable cases would provide at least 64 true bacteremia cases. This is consistent with established recommendations for external validation of clinical prediction models [31, 38] and allows estimation of sensitivity with a lower 95% confidence interval bound of approximately 0.94, given an expected point estimate of 0.99.

2.7 Statistical Analysis

Continuous variables are reported as median (interquartile range [IQR]) and were compared using the Mann-Whitney U test. Differences in medians were estimated using the Hodges-Lehmann estimator with 95% confidence intervals (CIs). Categorical variables are reported as number (percentage) and were compared using the Fisher exact test. Differences in proportions are reported with 95% CIs calculated using the Newcombe

method with continuity correction. For Gram-stain morphology, a single global comparison of the distribution of the six morphotypes between groups was performed using the Fisher-Freeman-Halton exact test. The four CDR predictors were applied to each case and the resulting risk classification was compared with the reference standard. Diagnostic accuracy was assessed by sensitivity, specificity, positive predictive value (PPV), and negative predictive value (NPV), with Clopper-Pearson exact 95% CIs. The proportion of true bacteremia within each TTP interval was reported with Wilson score 95% CIs. To assess abstraction reliability, a random 10% of charts was independently reviewed by a third investigator (CIM) blinded to the initial extraction. Interrater agreement was assessed using Cohen's κ for the two variables requiring interpretive judgment: the reference-standard outcome (true bacteremia versus contaminant) and suspected osteoarticular infection. The remaining predictors (Gram-stain result, TTP, indwelling device) were extracted directly from laboratory reports or medical records and were not subject to formal reliability assessment. As a secondary analysis, we estimated the antibiotic exposure that would have been avoided by applying the CDR to discharge low-risk cases without antibiotics, using observed prescribing patterns as the comparator. As a sensitivity analysis to assess the robustness of the rule to the choice of TTP threshold, we recalculated diagnostic accuracy while varying the TTP cutoff continuously from 12 to 24 hours, holding the three other predictors constant. Sensitivity, specificity, and predictive values with Clopper-Pearson exact 95% CIs were derived at each threshold and are presented graphically across the full range and tabulated at hourly increments. All tests were two-sided, and a P value less than 0.05 was considered statistically significant. No correction

for multiple testing was applied given the study's pre-specified primary endpoint. Analyses were performed using R version 4.6.0 (R Foundation for Statistical Computing).

3. Results

3.1 Study Population

From January 1, 2015 to May 31, 2025, 324 positive blood cultures were identified, and 130 evaluable cases were enrolled (Fig 2). Their demographic, clinical, and microbiological characteristics stratified by reference-standard outcome (true bacteremia versus contaminant) are presented in Table 1. Immunocompromised status did not differ significantly (15.4% vs 26.9%, $P = 0.137$), whereas indwelling devices and suspected osteoarticular infections were strongly associated with true bacteremia (both $P < 0.001$). C-reactive protein and procalcitonin were markedly higher in true bacteremia (both $P < 0.001$). Interrater agreement was $\kappa = 1.00$ for both the reference-standard outcome and the suspected osteoarticular infection predictor across the 13 randomly sampled duplicate charts. The corresponding characteristics of the original validation cohort [24] are presented in S3 Table.

Fig 2. Flow of participants through the validation cohort

Table 1. Baseline and Clinical Characteristics of Study Patients, by Reference-Standard Classification

Characteristic	Contaminants (N = 52)	True Bacteremia (N = 78)	Difference (95% CI)	*P* value
**Demographics**				
Sex, n (%)				0.717

Male	31 (59.6)	49 (62.8)		
Female	21 (40.4)	29 (37.2)		
Age, median (IQR), y	1.8 (0.1-7.7)	5.0 (1.6-10.8)	1.5 (0.2-3.1)	0.015
**Clinical features**				
Immunocompromised, n (%)	8 (15.4)	21 (26.9)	11.5% (-4.6-25.5)	0.137
Indwelling device, n (%)	1 (1.9)	24 (30.8)	28.8% (15.2-40.6)	<0.001
Suspected osteoarticular infection, n (%)	0 (0)	16 (20.5)	20.5% (8.8-31.5)	<0.001
**Laboratory values**				
C-Reactive Protein, median (IQR), mg/L	9.0 (2.8-59.5)	66.0 (32.1-102.0)	31.0 (12.7-50.5)	<0.001
Procalcitonin[b] median (IQR), µg/L	0.4 (0.1-0.8)	4.5 (3.4-76.3)	0.87 (0.23-3.45)	<0.001
**Gram stain[c]**				<0.001
Gram positive cocci in clusters, n (%)	36 (69.2)	30 (38.5)		
Gram positive cocci in chains, n (%)	6 (11.5)	20 (25.6)		
Gram positive cocci in pairs, n (%)	0 (0)	5 (6.4)		
Gram positive bacilli, n (%)	6 (11.5)	0 (0)		
Gram negative cocci, n (%)	0 (0)	1 (1.3)		
Gram negative bacilli, n (%)	4 (7.7)	22 (28.2)		

Abbreviations: CI, confidence interval; IQR, interquartile range; y, years.
[a]Differences in proportions are presented with 95% CIs calculated using the Newcombe method with continuity correction. Differences in medians are Hodges-Lehmann estimates with 95% CIs.
[b]Procalcitonin available in 36 of 52 contaminants and 56 of 78 true bacteremia cases.
[c]P value for Gram stain reflects a single global comparison of the distribution of the six morphotypes between groups, calculated using the Fisher-Freeman-Halton exact test.

3.2 Main results

The median TTP was 11.8 hours (IQR, 8.3-16.9) for true bacteremia and 22.8 hours (IQR 17.8-33.5) for contaminants, a difference of -11.0 hours (95% CI, -12.9 to -7.8). The proportion of true bacteremia declined as TTP increased, from 24 of 27 cases (88.9%; 95% CI, 71.9%-96.1%) within 10 hours to 3 of 14 (21.4%; 95% CI, 7.6%-47.6%) by 22 to 24 hours (Fig 3). Overall, 72 of 78 true bacteremias (92.3%; 95% CI, 84.2%-96.4%) were detected within 24 hours of sampling.

Fig 3. Distribution of bacteremia vs. contaminants according to TTP (N = 130)

3.3 Performance of the Sainte-Justine CDR

Of the 78 children with true bacteremia, the CDR classified 48 (61.5%) as high risk and 28 (35.9%) as moderate risk. Two were classified as low risk (false negatives). Of the 52 children with contaminants, 36 (69.2%) were correctly classified as low risk, while 10 (19.2%) were classified as high risk and 6 (11.5%) as moderate risk (false positives). Overall diagnostic performance is provided in Fig 4.

Fig 4. Risk stratification and diagnostic performance of the clinical decision rule for distinguishing true bacteremia from contaminants. (A) Distribution of cases across the three CDR risk strata. (low, moderate, high), shown separately for contaminants (N = 52) and true bacteremia (N = 78). Bars represent the within-group proportion, with absolute counts and percentages labeled. The gradient illustrates the rule's discrimination, with most contaminants classified as low risk and most true bacteremia as high risk. (B) Diagnostic accuracy of the rule with point estimates and 95% confidence intervals for sensitivity, specificity, positive predictive value, and negative predictive value. Confidence interval calculated by the Clopper-Pearson exact method. CDR, clinical decision rule; CI, confidence interval; NPV, negative predictive value; PPV, positive predictive value.

3.4 Threshold sensitivity analysis

Across the full range from 12 to 24 hours, sensitivity remained consistently high (94.9% to 98.7%), as did the negative predictive value (91.1% to 96.8%). As expected, raising the threshold increased sensitivity at the cost of specificity, which declined from 78.8% (95% CI, 65.3-88.9) at 14 hours to 57.7% (95% CI, 43.2-71.3) at 20 hours (Fig 5 and S4 Table). At the derivation threshold of 17 hours, sensitivity was 97.4% (95% CI, 91.0-99.7) and specificity 69.2% (95% CI, 54.9-81.3).

Fig 5. Sensitivity (green) and specificity (purple) of the clinical decision rule across time-to-positivity thresholds (12–24 hours). Shaded bands are 95% confidence intervals. The dashed line marks the 17-hour derivation threshold.

3.5 False negative cases

Both children misclassified as low risk had confirmed true bacteremia caused by methicillin-susceptible *Staphylococcus aureus*. One isolate was Panton-Valentine leukocidin-positive. Neither patient was immunocompromised or carried an indwelling device. In both, the Gram stain did not fulfil the high-risk morphology criterion, and TTP exceeded the 17-hour threshold (17.7 and 20.6 hours, respectively). These cases therefore represent borderline presentations in which classification was driven solely by a TTP value above the cut-off, with no other high-risk feature.

3.6 Potential impact on antibiotic exposure

In the contaminant group, 37 of 52 children (71.2%) received antibiotics under usual practice. Of these 37, 26 (70.3%) were classified as low risk by the CDR and represent potentially avoidable antibiotic exposure. Applying the rule would thus have reduced antibiotic exposure among contaminants from 71.2% to 21.2%, an absolute reduction of 50 percentage points, corresponding to one unnecessary antibiotic course potentially averted for every two contaminants to whom the rule was applied. The two false-negative cases received antibiotics regardless of CDR classification, reflecting the role of clinical judgement at the bedside.

4. Discussion

4.1 Main findings and interpretation

This first external validation of the Sainte-Justine CDR in a Swiss tertiary pediatric emergency cohort confirms strong diagnostic performance. The sensitivity of 97.4% is marginally lower than the 99% at derivation [24], consistent with the accuracy loss commonly seen when prediction models are transported to new settings [31, 38]. The specificity of 69.2% was higher than the 60% reported by Gravel et al. [24] and the 11% of the Hospital for Sick Children algorithm evaluated in the same Montreal cohort [11]. The management heterogeneity reported in a recent Canadian survey [18] reflects a genuine unmet need for structured decision support.

4.2 Interpretation of false negatives

The two missed cases warrant scrutiny, as sensitivity is the principal safety property of a rule that defers antibiotics. Both occurred in immunocompetent children without an indwelling device, and in both the Gram stain did not fulfil the high-risk morphology criterion, with. TTP just above the threshold. These values are consistent with published pediatric distributions for *Staphylococcus aureus* bacteremia, which extend well beyond 17 hours and are substantially longer than for pathogens such as *Escherichia coli* or group B *Streptococcus* [39, 40]. Both cases are therefore microbiologically plausible as true bacteremia, although no firm conclusions can be drawn from two observations. Critically, neither presented any other high-risk feature that could have served as a safety signal at culture reporting. This argues for pairing rule-based antibiotic deferral with a structured

24-hour clinical and microbiological safety net, rather than pursuing threshold refinement alone.

The TTP predictor depends on pre-analytical conditions. Delayed loading of bottles held at room temperature substantially shortens TTP [32]. TTP is therefore modulated by transport time, holding temperature, and laboratory hours, rather than being solely intrinsic to the organism. At our institution, short transport times and infrequent delayed loading made such shortening unlikely [32], but the 17-hour threshold may not transfer unchanged across laboratories with different pre-analytical workflows, supporting local calibration in any prospective implementation. The practical impact of this dependence, however, hinges on how much the rule's accuracy actually shifts when the threshold is varied, which we examined directly.

4.3 Clinical decision rule robustness

Our sensitivity analysis showed that performance was stable across TTP thresholds from 12 to 24 hours, with sensitivity remaining above 94% throughout. This robustness is clinically reassuring, because the optimal TTP cutoff reported in the literature varies appreciably with the population, the blood culture system, and the era of testing. Recent studies have proposed thresholds of approximately 16 to 20 hours, reflecting the shorter detection times of modern automated systems [41, 42]. The derivation threshold of 17 hours falls within this range, and the absence of any abrupt change around it indicates that the rule does not depend on precise calibration to a single institution's culture system. This is consistent with the underlying biology. Most bacteremias are detected early, with

roughly 90% positive by 24 hours [39], so the rule retains high sensitivity even when the threshold is lowered.

4.4 Molecular diagnostics and the persistent need for contaminant discrimination

The growing availability of rapid molecular diagnostics might appear to reduce the need for a clinical rule. These assays, however, address a different question. These panels accelerate organism identification and antimicrobial optimization once a culture is positive, but do not determine whether the detected organism represents true infection or contamination [43]. Their higher analytical sensitivity may in fact even increase detection of contaminant DNA. In one pediatric study, PCR yielded contaminant results in 22% of samples versus 6% for culture [44]. A recent systematic review and meta-analysis found that rapid molecular assays still lack the sensitivity to replace blood culture as a standalone diagnostic [45]. Their diagnostic performance improves when interpreted against a composite clinical diagnosis [46, 47]. The task of discriminating true bacteremia from contaminants therefore remains fundamentally clinical, and a structured rule complements rather than competes with molecular methods.

4.5 Comparison with previous works

Compared with the Hospital for Sick Children algorithm [11], the Sainte-Justine CDR achieves a more favorable balance between sensitivity and specificity, largely because the earlier algorithm classified most contaminants as bacteremia. The higher specificity reflects in part the inclusion of TTP as a predictor, consistent with the reported association

between rapid positivity and true bacteremia [25]. These comparisons warrant caution, as sensitivity and specificity remain sensitive to differences in case mix and severity across cohorts, and the TTP threshold is itself subject to pre-analytical variability [48].

Our findings also align with evidence on the limitations of biomarkers as standalone discriminators. Neither C-reactive protein nor procalcitonin, nor composite tools such as the Lab-score were designed for the binary task of distinguishing true bacteremia from contamination. Both perform moderately well for severe bacterial infection, but were validated in broader febrile populations rather than in children with a preliminary positive blood culture [28, 29, 49]. Structured rules combining microbiological, clinical and host predictors are therefore more likely than biomarkers alone to support management changes in this scenario.

4.6 Clinical implications

If reproduced prospectively, the rule could reduce unnecessary antibiotic exposure while preserving the safety net for true bacteremia. In our cohort, 71% of children with contaminant cultures received antibiotics under usual practice. Half of all contaminants (26 of 52) were both treated and classified as low risk by the CDR, representing antibiotic courses that could potentially have been avoided. Antibiotic stewardship in pediatric sepsis workup remains a recognized priority, and structured discrimination of positive blood cultures offers a concrete entry point for bedside stewardship.

4.7 Limitations

This study has several limitations. First, the reference standard was not fully independent from the predictors included in the CDR, particularly Gram-stain morphology and clinical suspicion of osteoarticular infection, creating a risk of incorporation bias that may have overstated observed diagnostic performance. But no perfect reference standard exists for contamination. These risks were mitigated by blinded sequential adjudication, two independent reviewers, and a structured case report form. Second, the specialist's assessment incorporated the Gram-stain result and organism identity, which also inform the CDR’s high-risk gate, raising potential incorporation bias for that predictor. However, TTP, the rule's most discriminant predictor, was not available to clinicians at the bedside and could not have influenced the reference standard. Third, the sample size of 130 evaluable cases yields acceptable but relatively wide confidence intervals, particularly for specificity, although the sensitivity analysis confirmed that diagnostic performance was robust across TTP thresholds from 12 to 24 hours. Fourth, single-center recruitment limits generalizability. Fifth, the antibiotic exposure estimate reflects observed prescribing patterns rather than prospective behavior, and random case selection minimizes selection bias. Finally, in our cohort, 61 of 130 cultures (46.9%) were collected outside laboratory operating hours and remained at room temperature before loading into the continuous-monitoring automated incubation systems, potentially resulting in shorter measured TTP values. As this effect would shift cases toward the high-risk side of the 17-hour threshold, it would be expected to maintain rather than compromise the rule’s sensitivity [32].

5. Conclusion and perspectives

This first external validation of the Sainte-Justine CDR supports its diagnostic performance. The rule offers a more favorable balance between sensitivity and specificity than the Hospital for Sick Children algorithm and could reduce unnecessary antibiotic exposure in pediatric emergency settings. Prospective multicenter validation within pediatric emergency research networks is the next step. Impact studies should quantify changes in antibiotic prescribing, length of stay, return visits, and missed bacteremia, and determine whether the TTP threshold requires local calibration given institutional differences in pre-analytical workflows, and whether a structured 24-hour follow-up pathway is needed when antibiotics are deferred.

6. Acknowledgements

We thank the PIEUVRE (Program for the Integration of Early-career University student Volunteers in RESearch) team at Geneva University Hospitals for their support throughout this work, and the microbiology laboratory of HUG for facilitating data extraction.

7. References

1. Cioffredi LA, Jhaveri R. Evaluation and management of febrile children: a review. JAMA Pediatr. 2016;170(8):794-800. Epub 2016/06/21. doi: 10.1001/jamapediatrics.2016.0596. PubMed PMID: 27322346.
2. Shapiro DJ, Fine AM. Patient ethnicity and pediatric visits to the emergency department for fever. Pediatr Emerg Care. 2021;37(11):555-9. Epub 2019/11/13. doi: 10.1097/PEC.0000000000001945. PubMed PMID: 31714448.
3. Norberg A, Christopher NC, Ramundo ML, Bower JR, Berman SA. Contamination rates of blood cultures obtained by dedicated phlebotomy vs intravenous catheter. JAMA. 2003;289(6):726-9. Epub 2003/02/15. doi: 10.1001/jama.289.6.726. PubMed PMID: 12585951.
4. Pantell RH, Roberts KB, Adams WG, Dreyer BP, Kuppermann N, O'Leary ST, et al. Evaluation and management of well-appearing febrile infants 8 to 60 days old. Pediatrics. 2021;148(2). Epub 2021/07/21. doi: 10.1542/peds.2021-052228. PubMed PMID: 34281996.
5. Leazer RC. Evaluation and management of young febrile infants: an overview of the new AAP guideline. Pediatr Rev. 2023;44(3):127-38. Epub 2023/03/01. doi: 10.1542/pir.2022-005624. PubMed PMID: 36854834.
6. Miller JM, Binnicker MJ, Campbell S, Carroll KC, Chapin KC, Gonzalez MD, et al. Guide to utilization of the microbiology laboratory for diagnosis of infectious diseases: 2024 update by the Infectious Diseases Society of America (IDSA) and the American Society for Microbiology (ASM). Clin Infect Dis. 2024. Epub 2024/03/05. doi: 10.1093/cid/ciae104. PubMed PMID: 38442248.
7. Gangoiti I, Zubizarreta A, Elgoibar B, Mintegi S, Infectious Diseases Working Group, Spanish Society of Pediatric Emergencies. Occult bacteremia in young children with very high fever without a source: a multicenter study. Pediatr Infect Dis J. 2020;39(12):e462-e4. Epub 2020/09/09. doi: 10.1097/INF.0000000000002891. PubMed PMID: 32898089.
8. Lane RD, Richardson T, Scott HF, Paul RM, Balamuth F, Eisenberg MA, et al. Delays to antibiotics in the emergency department and risk of mortality in children with sepsis. JAMA Netw Open. 2024;7(6):e2413955. Epub 2024/06/05. doi: 10.1001/jamanetworkopen.2024.13955. PubMed PMID: 38837160.
9. Watson RS, Carrol ED, Carter MJ, Kissoon N, Ranjit S, Schlapbach LJ. The burden and contemporary epidemiology of sepsis in children. Lancet Child Adolesc Health. 2024;8(9):670-81. Epub 2024/08/15. doi: 10.1016/S2352-4642(24)00140-8. PubMed PMID: 39142741.
10. Weiss SL, Fitzgerald JC. Pediatric sepsis diagnosis, management, and sub-phenotypes. Pediatrics. 2024;153(1). Epub 2023/12/12. doi: 10.1542/peds.2023-062967. PubMed PMID: 38084084.
11. Gravel J, Grandjean-Blanchet C, Demean-Loghin A, Noyon B, Ostrow O, Vallieres E. Validation of the Hospital for Sick Children algorithm for discriminating bacteremia from contaminants in children with a preliminary positive blood culture. Ann Emerg Med. 2024;84(5):490-9. Epub 2024/06/18. doi: 10.1016/j.annemergmed.2024.05.005. PubMed PMID: 38888530.

12. Segal GS, Chamberlain JM. Resource utilization and contaminated blood cultures in children at risk for occult bacteremia. Arch Pediatr Adolesc Med. 2000;154(5):469-73. Epub 2000/05/12. doi: 10.1001/archpedi.154.5.469. PubMed PMID: 10807297.
13. El Feghaly RE, Chatterjee J, Dowdy K, Stempak LM, Morgan S, Needham W, et al. A quality improvement initiative: reducing blood culture contamination in a children's hospital. Pediatrics. 2018;142(4). Epub 2018/09/16. doi: 10.1542/peds.2018-0244. PubMed PMID: 30217808.
14. Megged O, Dorembus S, Ben-Shalom E, Heiman E. Comparing blood culture contamination rates by different sampling methods in a pediatric emergency department. J Paediatr Child Health. 2022;58(4):669-73. Epub 2021/10/21. doi: 10.1111/jpc.15812. PubMed PMID: 34668594.
15. Dempsey C, Skoglund E, Muldrew KL, Garey KW. Economic health care costs of blood culture contamination: a systematic review. Am J Infect Control. 2019;47(8):963-7. Epub 2019/02/24. doi: 10.1016/j.ajic.2018.12.020. PubMed PMID: 30795840.
16. Murofushi Y, Furuichi M, Shoji K, Kubota M, Ishiguro A, Uematsu S, et al. Adverse economic impact associated with blood culture contamination in a pediatric emergency department. Pediatr Infect Dis J. 2018;37(8):755-8. Epub 2018/05/31. doi: 10.1097/INF.0000000000001898. PubMed PMID: 29846358.
17. Indarwati F, Mathew S, Munday J, Keogh S. Incidence of peripheral intravenous catheter failure and complications in pediatric patients: systematic review and meta analysis. Int J Nurs Stud. 2020;102:103488. Epub 2019/12/22. doi: 10.1016/j.ijnurstu.2019.103488. PubMed PMID: 31862530.
18. Gouin S, Carriere B, Gravel J. Management of a positive blood culture in the pediatric emergency department: a multicenter case-based survey. CJEM. 2025. Epub 2025/04/16. doi: 10.1007/s43678-025-00908-3. PubMed PMID: 40234376.
19. Kuppermann N, Dayan PS, Levine DA, Vitale M, Tzimenatos L, Tunik MG, et al. A clinical prediction rule to identify febrile infants 60 days and younger at low risk for serious bacterial infections. JAMA Pediatr. 2019;173(4):342-51. Epub 2019/02/19. doi: 10.1001/jamapediatrics.2018.5501. PubMed PMID: 30776077.
20. Vaugon E, Dien Bard J. Revisiting diagnostics: multiplex infectious disease panels. Clin Microbiol Infect. 2026;32(2):203-6. Epub 2025/11/06. doi: 10.1016/j.cmi.2025.10.026. PubMed PMID: 41192794.
21. Lewinski MA, Alby K, Babady NE, Butler-Wu SM, Bard JD, Greninger AL, et al. Exploring the utility of multiplex infectious disease panel testing for diagnosis of infection in different body sites: a joint report of the Association for Molecular Pathology, American Society for Microbiology, Infectious Diseases Society of America, and Pan American Society for Clinical Virology. J Mol Diagn. 2023;25(12):857-75. Epub 2023/09/28. doi: 10.1016/j.jmoldx.2023.08.005. PubMed PMID: 37757952.
22. Candel FJ, Salavert M, Canton R, Del Pozo JL, Galan-Sanchez F, Navarro D, et al. The role of rapid multiplex molecular syndromic panels in the clinical management of infections in critically ill patients: an experts-opinion document. Crit Care. 2024;28(1):440. Epub 2024/12/31. doi: 10.1186/s13054-024-05224-3. PubMed PMID: 39736683.
23. Ramanan P, Bryson AL, Binnicker MJ, Pritt BS, Patel R. Syndromic panel-based testing in clinical microbiology. Clin Microbiol Rev. 2018;31(1). Epub 2017/11/17. doi: 10.1128/CMR.00024-17. PubMed PMID: 29142077.

24. Gravel J, Grandjean-Blanchet C, Loghin AD, Noyon B, Ostrow O, Vallieres E, et al. Derivation and validation of a clinical rule to detect bacteremia versus contaminants in positive pediatric blood cultures: a retrospective cohort study. Ann Emerg Med. 2025;86(6):576-85. Epub 2025/08/14 22:52. doi: 10.1016/j.annemergmed.2025.06.620. PubMed PMID: 40810705.
25. Noyon B, Loghin AD, Grandjean-Blanchet C, Ostrow O, Vallières É, Gravel J. Association between time to gram stain positivity and true bacteremia in pediatric emergency department patients. The American Journal of Emergency Medicine. 2025. doi: https://doi.org/10.1016/j.ajem.2025.05.019.
26. Guo BC, Chen YT, Chang YJ, Chen CY, Lin WY, Wu HP. Predictors of bacteremia in febrile infants under 3 months old in the pediatric emergency department. BMC Pediatr. 2023;23(1):444. Epub 2023/09/08. doi: 10.1186/s12887-023-04271-z. PubMed PMID: 37679686.
27. Mannino Avila E, Stucky Fisher E, Rhee K. True versus false bacteremia in infants and children less than 3 years of age. Pediatr Emerg Care. 2021;37(6):e307-e12. Epub 2018/08/15. doi: 10.1097/PEC.0000000000001585. PubMed PMID: 30106869.
28. Chiu IM, Huang YH, Su CM, Kung CT, Li CJ, Chen CH, et al. C-reactive protein concentration can help to identify bacteremia in children visiting the emergency department: a single medical center experience. Pediatr Emerg Care. 2020;36(6):291-5. Epub 2018/03/07. doi: 10.1097/PEC.0000000000001453. PubMed PMID: 29509648.
29. Norman-Bruce H, Umana E, Mills C, Mitchell H, McFetridge L, McCleary D, et al. Diagnostic test accuracy of procalcitonin and C-reactive protein for predicting invasive and serious bacterial infections in young febrile infants: a systematic review and meta-analysis. Lancet Child Adolesc Health. 2024;8(5):358-68. Epub 2024/03/19. doi: 10.1016/S2352-4642(24)00021-X. PubMed PMID: 38499017.
30. Collins GS, Moons KGM, Dhiman P, Riley RD, Beam AL, Van Calster B, et al. TRIPOD+AI statement: updated guidance for reporting clinical prediction models that use regression or machine learning methods. BMJ. 2024;385:e078378. Epub 2024/04/17. doi: 10.1136/bmj-2023-078378. PubMed PMID: 38626948.
31. Moons KG, Altman DG, Reitsma JB, Ioannidis JP, Macaskill P, Steyerberg EW, et al. Transparent Reporting of a multivariable prediction model for Individual Prognosis or Diagnosis (TRIPOD): explanation and elaboration. Ann Intern Med. 2015;162(1):W1-73. Epub 2015/01/07. doi: 10.7326/M14-0698. PubMed PMID: 25560730.
32. Cherkaoui A, Fischer A, Camus L, Crudeli L, Calderon DJ, Schrenzel J. Impact of delayed blood culture bottles incubation on the recovery and detection of microorganisms: effect of ambient room temperature versus 4 degrees C. Diagn Microbiol Infect Dis. 2026;115(2):117342. Epub 2026/03/01. doi: 10.1016/j.diagmicrobio.2026.117342. PubMed PMID: 41763125.
33. World Medical Association. World Medical Association declaration of Helsinki: ethical principles for medical research involving human participants. JAMA. 2025;333(1):71-4. Epub 2024/10/19 20:42. doi: 10.1001/jama.2024.21972. PubMed PMID: 39425955.
34. Bossuyt PM, Reitsma JB, Bruns DE, Gatsonis CA, Glasziou PP, Irwig L, et al. STARD 2015: an updated list of essential items for reporting diagnostic accuracy studies. BMJ. 2015;351:h5527. Epub 2015/10/30. doi: 10.1136/bmj.h5527. PubMed PMID: 26511519.

35. von Elm E, Altman DG, Egger M, Pocock SJ, Gotzsche PC, Vandenbroucke JP, et al. The Strengthening the Reporting of Observational Studies in Epidemiology (STROBE) statement: guidelines for reporting observational studies. Lancet. 2007;370(9596):1453-7. Epub 2007/12/08. doi: 10.1016/S0140-6736(07)61602-X. PubMed PMID: 18064739.

36. CDC Centers for Disease Control and Prevention. NHSN organism list (all organisms, common commensals, MBI organisms, and UTI bacteria) Atlanta, GA, National Healthcare Safety Network (NHSN); 2004. Available from: https://www.cdc.gov/nhsn/xls/master-organism-com-commensals-lists.xlsx.

37. Doern GV, Carroll KC, Diekema DJ, Garey KW, Rupp ME, Weinstein MP, et al. Practical guidance for clinical microbiology laboratories: a comprehensive update on the problem of blood culture contamination and a discussion of methods for addressing the problem. Clin Microbiol Rev. 2019;33(1). Epub 2019/11/02. doi: 10.1128/CMR.00009-19. PubMed PMID: 31666280.

38. Collins GS, Reitsma JB, Altman DG, Moons KG. Transparent reporting of a multivariable prediction model for individual prognosis or diagnosis (TRIPOD): the TRIPOD statement. BMJ. 2015;350:g7594. Epub 2015/01/09. doi: 10.1136/bmj.g7594. PubMed PMID: 25569120.

39. Biondi EA, Mischler M, Jerardi KE, Statile AM, French J, Evans R, et al. Blood culture time to positivity in febrile infants with bacteremia. JAMA Pediatr. 2014;168(9):844-9. Epub 2014/07/23. doi: 10.1001/jamapediatrics.2014.895. PubMed PMID: 25048522.

40. Thornley EM, Alexander R, Popelka J, Montgomery CP. Risk factors for persistent Staphylococcus aureus bacteremia in children. Pediatr Infect Dis J. 2024;43(11):1040-5. Epub 2024/06/21. doi: 10.1097/INF.0000000000004439. PubMed PMID: 38905222.

41. Manabe Y, Hagiya H, Fukushima S, Nakamoto K, Oguni K, Akazawa H, et al. Time to positivity for differentiating blood culture contamination: a 20-hour cutoff for major contaminants. Diagn Microbiol Infect Dis. 2025;113(4):117030. Epub 2025/08/06. doi: 10.1016/j.diagmicrobio.2025.117030. PubMed PMID: 40768822.

42. Laque-Ale A, Hueda-Zavaleta M, Gomez de la Torre JC, Alvarado L, Caceres Del Aguila JA. Diagnostic performance of the time to positivity of blood cultures to distinguish true bacteremia from contaminants based on an automated system. Rev Peru Med Exp Salud Publica. 2023;40(4):451-8. Epub 2023/01/01. doi: 10.17843/rpmesp.2023.404.12724. PubMed PMID: 38597473.

43. Caspar Y, Deves A, Richarme C, Le Marechal M, Ponderand L, Mounayar AL, et al. Correction to: Clinical impact and cost-consequence analysis of ePlex(R) blood culture identification panels for the rapid diagnosis of bloodstream infections: a single-center randomized controlled trial. Eur J Clin Microbiol Infect Dis. 2024;43(11):2245-6. Epub 2024/08/31. doi: 10.1007/s10096-024-04924-6. PubMed PMID: 39207590.

44. Checa RMC, Gijon M, Hofheinz SB, Rojo P. Multiple polymerase chain reaction for direct detection of bloodstream infection after cardiac surgery in a PICU. Crit Care Explor. 2022;4(6):e0707. Epub 2022/06/03. doi: 10.1097/CCE.0000000000000707. PubMed PMID: 35651736.

45. Rapszky GA, Do To UN, Kiss VE, Koi T, Walter A, Gergo D, et al. Rapid molecular assays versus blood culture for bloodstream infections: a systematic review and meta-analysis. EClinicalMedicine. 2025;79:103028. Epub 2025/02/19. doi: 10.1016/j.eclinm.2024.103028. PubMed PMID: 39968206.

46. Wu J, Tang B, Qiu Y, Tan R, Liu J, Xia J, et al. Clinical validation of a multiplex droplet digital PCR for diagnosing suspected bloodstream infections in ICU practice: a promising diagnostic tool. Crit Care. 2022;26(1):243. Epub 2022/08/09. doi: 10.1186/s13054-022-04116-8. PubMed PMID: 35941654.
47. Andrei AI, Talapan D, Rafila A, Popescu GA. Influence of multiplex PCR in the management of antibiotic treatment in patients with bacteremia. Antibiotics (Basel). 2023;12(6). Epub 2023/06/28. doi: 10.3390/antibiotics12061038. PubMed PMID: 37370357.
48. Whittington KJ, Malone SM, Hogan PG, Ahmed F, Flowers J, Milburn G, et al. Staphylococcus aureus bacteremia in pediatric patients: uncovering a rural health challenge. Open Forum Infect Dis. 2023;10(7):ofad296. Epub 2023/07/20. doi: 10.1093/ofid/ofad296. PubMed PMID: 37469617.
49. Lacour AG, Zamora SA, Gervaix A. A score identifying serious bacterial infections in children with fever without source. Pediatr Infect Dis J. 2008;27(7):654-6. Epub 2008/06/10. doi: 10.1097/INF.0b013e318168d2b4. PubMed PMID: 18536624.

8. Supporting information

S1 Table. Indwelling devices

S2 Table. Data Extraction

S3 Table. Baseline and Clinical Characteristics of the Sainte-Justine Derivation Cohort

S4 Table. Diagnostic Accuracy of the Clinical Decision Rule Across Time-to-Positivity Thresholds From 12 to 24 Hours

Figure 1

Click here to access/download;Figure;Fig. 1.tiff

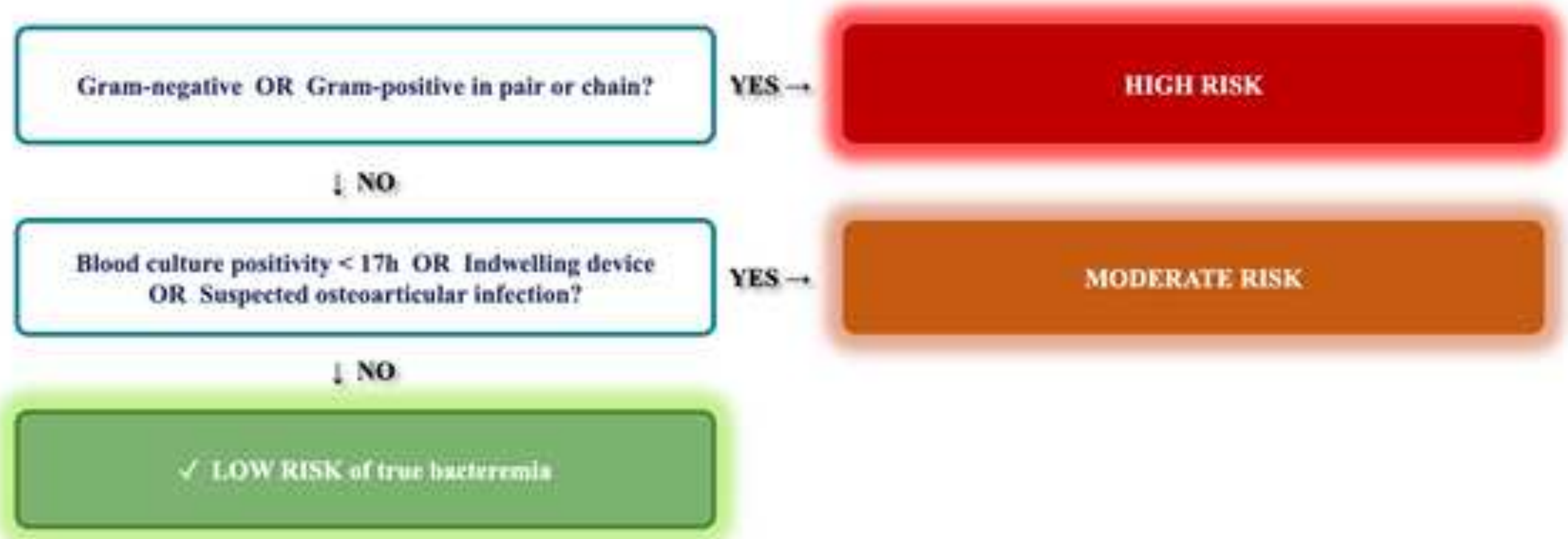

Figure 2

Click here to access/download;Figure;Fig. 2.tiff

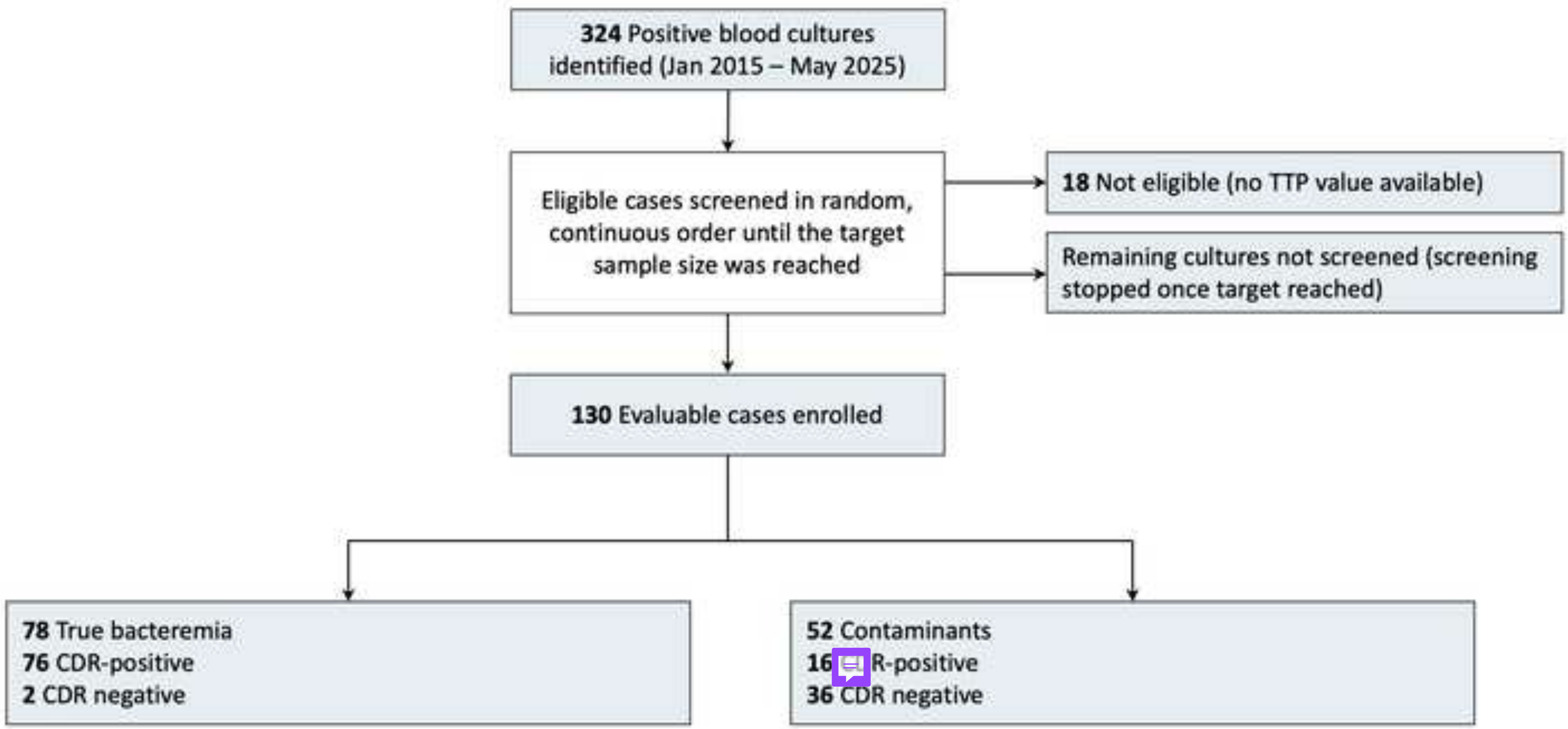

Figure 3

Click here to access/download;Figure;Fig. 3.tiff

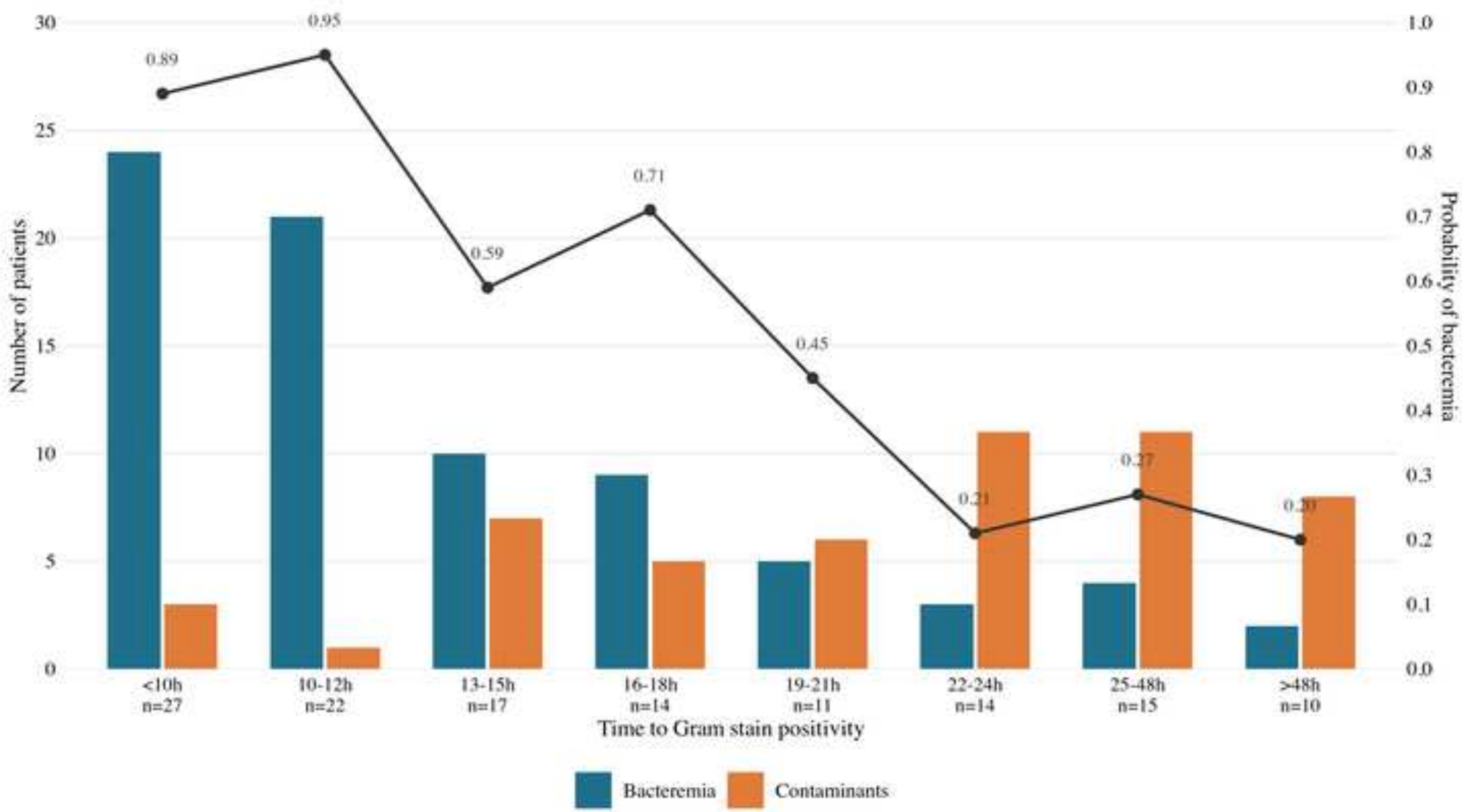

Figure 4

Click here to access/download;Figure;Fig. 4.tiff

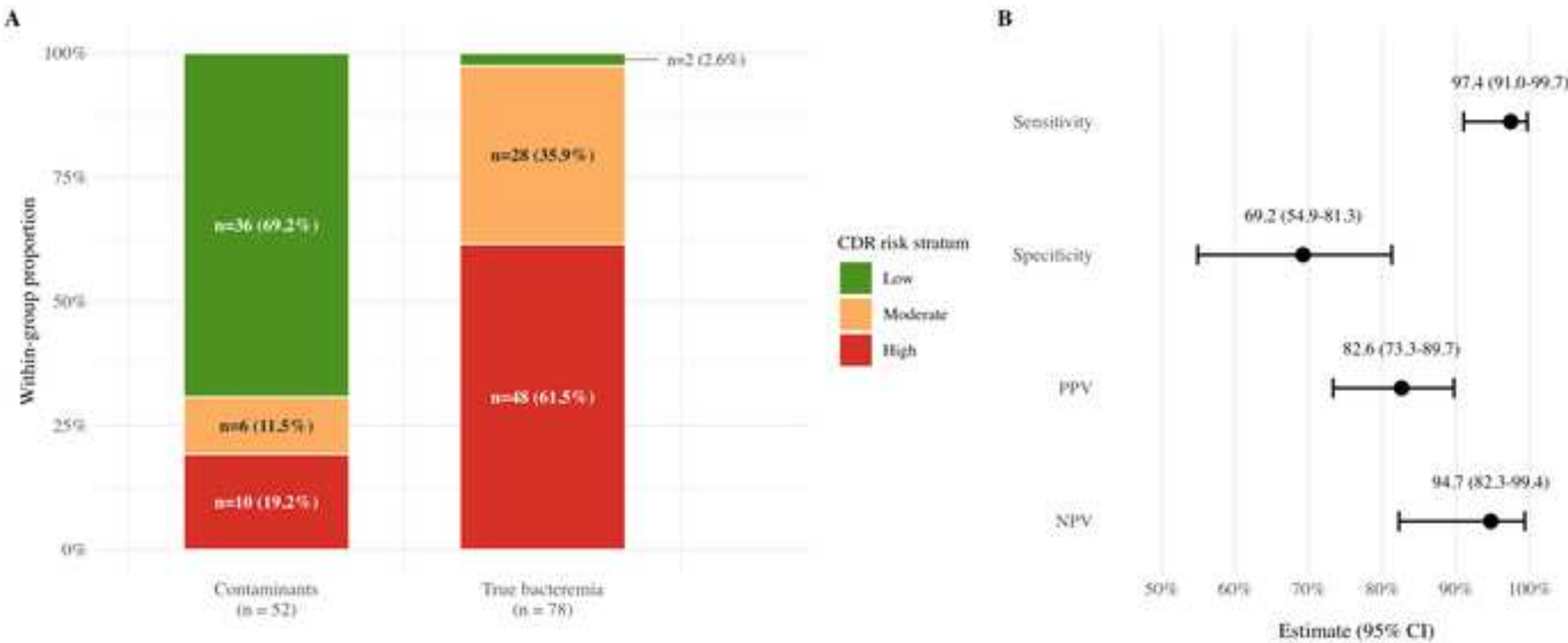

Figure 5

Click here to access/download;Figure;Fig. 5.tiff

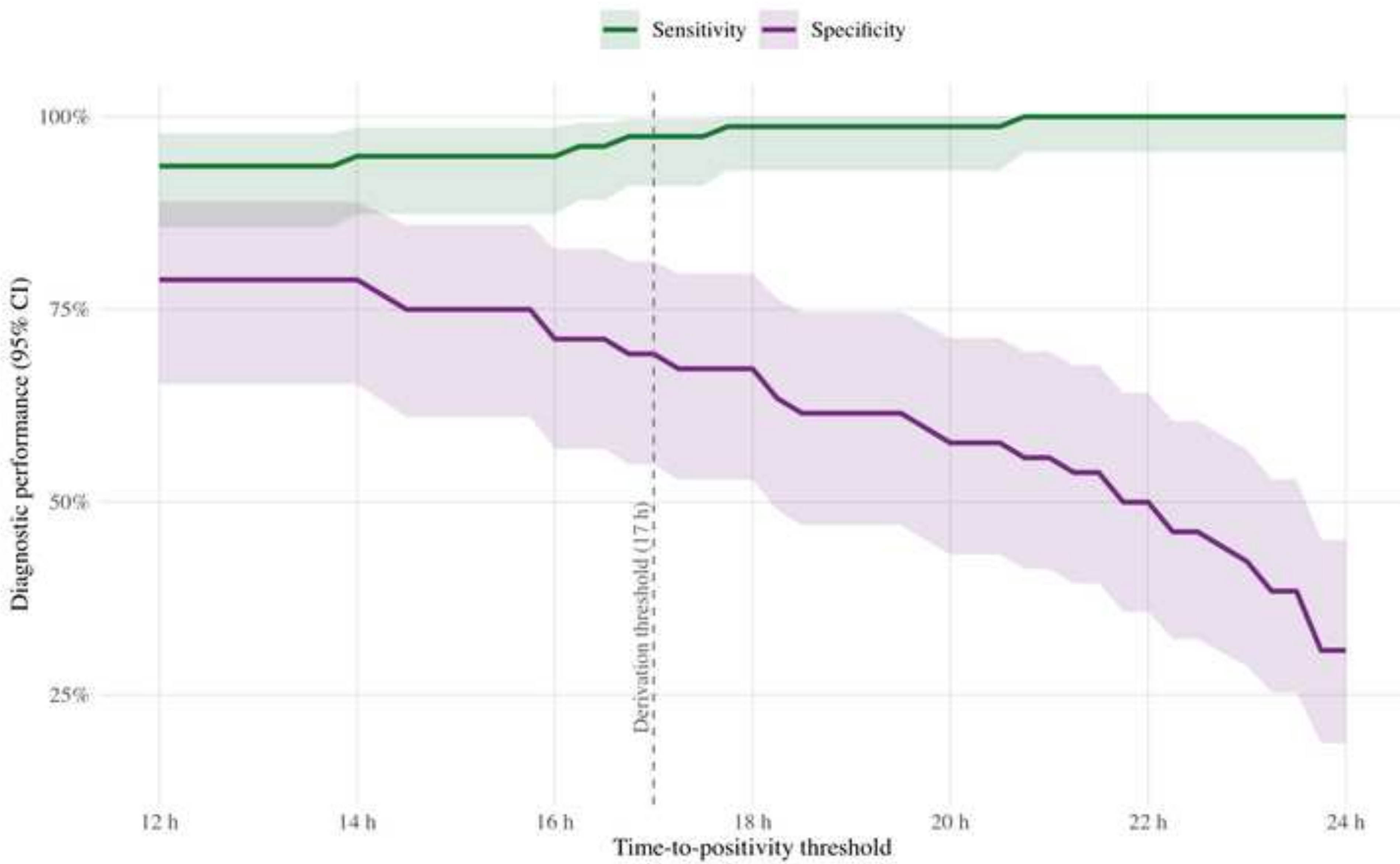

Click here to access/download
Supporting Information
S1_File.docx
