# Supplementary material for: External validation of a decision rule for bacteremia vs contaminants in pediatric blood cultures
Source: PLoS One. 2026 Nov 13;21(11):e0355010. doi: 10.1371/journal.pone.0355010 (PMC13766231; doi:10.1371/journal.pone.0355010)
Supplement: Supplementary file 2 [file pone.0355010.s001.docx]

**S1 Table. Indwelling devices**

| **Category** | **Device** |
| --- | --- |
| Central venous access | Non-tunneled central venous catheter |
|  | Peripherally inserted central catheter (PICC) |
|  | Tunneled catheter (Broviac, Hickman) |
|  | Totally implantable venous access port |
|  | Hemodialysis catheter |
| Cerebrospinal fluid diversion | Ventriculoperitoneal shunt |
|  | External ventricular drain |

A case met the indwelling-device predictor if any listed device was present at the index visit
