# Supplementary material for: External validation of a decision rule for bacteremia vs contaminants in pediatric blood cultures
Source: PLoS One. 2026 Nov 13;21(11):e0355010. doi: 10.1371/journal.pone.0355010 (PMC13766231; doi:10.1371/journal.pone.0355010)
Supplement: Supplementary file 3 [file pone.0355010.s002.docx]

**S2 Table. Data Extraction**

| **Category** | **Variable** | **Definition / coding** | **Data source** |
| --- | --- | --- | --- |
| Demographics | Age | Age in years at index visit | EHR |
|  | Sex | Male / female | EHR |
|  | Index visit date | DD/MM/YYYY | EHR |
| Comorbidities | Immunosuppression | Present / absent (per documented diagnosis or therapy) | EHR |
|  | Indwelling device^a^ | Present / absent at index visit (S1 Table) | EHR |
| Presenting features | Fever at index visit | Documented temperature ≥38.0 °C or reported fever: yes / no | EHR |
|  | Musculoskeletal complaints | Documented: yes / no | EHR |
|  | Suspected osteoarticular infection^a^ | Suspected septic arthritis or osteomyelitis at index visit: yes / no | EHR |
| Laboratory | C-reactive protein | mg/L | Laboratory record |
|  | Procalcitonin | µg/L | Laboratory record |
| Microbiological | Organism identified | Species / genus per laboratory identification | Microbiology database |
|  | Gram stain morphology^a^ | Gram-negative bacilli; Gram-positive cocci in pairs, chains, or clusters; etc. | Microbiology database |
|  | Time to positivity^a^ | Hours:Minutes from bottle placement into BD BACTEC FX to growth detection | Microbiology database |
|  | Repeat blood culture results | Positive / negative / not performed | Microbiology database |
| Management | Antibiotic administration | Empirical antibiotic given: yes / no | EHR |
|  | Antibiotic regimen | INN(s) of antibiotic(s) administered | EHR |
|  | Hospitalization | Admitted / discharged from PED | EHR |
|  | Follow-up status | Documented outcome at follow-up | EHR |
|  | PID specialist assessment | Documented assessment by pediatric infectious disease specialist | EHR |
| Outcome | True bacteremia^b^ | True bacteremia / contaminant, by two-step independent adjudication | Adjudication (see Outcome) |

EHR, Electronic Health Record; CVC, Central Venous Catheter; PICC, Peripherally Inserted Central Catheter; PID, Pediatric Infectious Disease; PED, Pediatric Emergency Department; INN, International Non-proprietary Name

^a^ Clinical decision rule predictor

^b^ Reference-standard outcome
