# Supplementary material for: External validation of a decision rule for bacteremia vs contaminants in pediatric blood cultures
Source: PLoS One. 2026 Nov 13;21(11):e0355010. doi: 10.1371/journal.pone.0355010 (PMC13766231; doi:10.1371/journal.pone.0355010)
Supplement: Supplementary file 4 [file pone.0355010.s003.docx]

**S3 Table. Baseline and Clinical Characteristics of the Sainte-Justine Derivation Cohort**

| **Characteristic** | **Contaminants**  (N = 90) | **True Bacteremia**  (N = 83) |
| --- | --- | --- |
| **Demographics** | | |
| Sex, n (%) | | |
| Male | 45 (50) | 49 (59) |
| Female | 45 (50) | 34 (41.0) |
| Age, median (IQR), y | 2.0 (0.3-5.9) | 2.7 (0.9-8.2) |
| **Clinical features** | | |
| Immunocompromised, n (%) | 7 (8) | 10 (12) |
| Indwelling device, n (%) | 9 (10) | 19 (23) |
| Suspected osteoarticular infection, n (%) | 2 (2) | 15 (18) |
| **Laboratory values** | | |
| C-Reactive Protein, median (IQR), mg/L | 9 (1-35) | 99 (46-180) |
| Procalcitonin^a^ median (IQR), µg/L | NA | NA |
| **Gram stain** | | |
| Gram positive cocci in clusters, n (%) | 53 (59) | 28 (34) |
| Gram positive cocci in chains, n (%) | 13 (14) | 20 (24) |
| Gram positive cocci in pairs, n (%) | 3 (3) | 3 (4) |
| Gram positive bacilli, n (%) | 18 (20) | 0 (0) |
| Gram negative cocci, n (%) | NA | NA |
| Gram negative bacilli, n (%) | 18 (20) | 34 (41) |
| Others | 2 (2) | 0 (0) |

Adapted from Gravel et al. [1]

**Reference**

1.	Gravel J, Grandjean-Blanchet C, Loghin AD, Noyon B, Ostrow O, Vallieres E, et al. Derivation and validation of a clinical rule to detect bacteremia versus contaminants in positive pediatric blood cultures: a retrospective cohort study. Ann Emerg Med. 2025;86(6):576-85. Epub 2025/08/14 22:52. doi: 10.1016/j.annemergmed.2025.06.620. PubMed PMID: 40810705.
