# Supplementary material for: External validation of a decision rule for bacteremia vs contaminants in pediatric blood cultures
Source: PLoS One. 2026 Nov 13;21(11):e0355010. doi: 10.1371/journal.pone.0355010 (PMC13766231; doi:10.1371/journal.pone.0355010)
Supplement: Supplementary file 5 [file pone.0355010.s004.docx]

**S4 Table. Diagnostic Accuracy of the Clinical Decision Rule Across Time-to-Positivity Thresholds From 12 to 24 Hours**

| **TTP Threshold** | **Sensitivity**  **(95% CI)** | **Specificity**  **(95% CI)** | **PPV**  **(95% CI)** | **NPV**  **(95% CI)** |
| --- | --- | --- | --- | --- |
| 12h | 93.6 (85.7-97.9) | 78.8 (65.3-88.9) | 86.9 (77.8-93.3) | 89.1 (76.4-96.4) |
| 13h | 93.6 (85.7-97.9) | 78.8 (65.3-88.9) | 86.9 (77.8-93.3) | 89.1 (76.4-96.4) |
| 14h | 94.9 (87.4-98.6) | 78.8 (65.3-88.9) | 87.1 (78.0-93.4) | 91.1 (78.8-97.5) |
| 15h | 94.9 (87.4-98.6) | 75.0 (61.1-86.0) | 85.1 (75.8-91.8) | 90.7 (77.9-97.4) |
| 16h | 94.9 (87.4-98.6) | 71.2 (56.9-82.9) | 83.1 (73.7-90.2) | 90.2 (76.9-97.3) |
| **17h** | **97.4 (91.0-99.7)** | **69.2 (54.9-81.3)** | **82.6 (73.3-89.7)** | **94.7 (82.3-99.4)** |
| 18h | 98.7 (93.1-100.0) | 67.3 (52.9-79.7) | 81.9 (72.6-89.1) | 97.2 (85.5-99.9) |
| 19h | 98.7 (93.1-100.0) | 61.5 (47.0-74.7) | 79.4 (70.0-86.9) | 97.0 (84.2-99.9) |
| 20h | 98.7 (93.1-100.0) | 57.7 (43.2-71.3) | 77.8 (68.3-85.5) | 96.8 (83.3-99.9) |
| 21 | 100.0 (95.4-100.0) | 55.8 (41.3-69.5) | 77.2 (67.8-85.0) | 100.0 (88.1-100.0) |
| 22h | 100.0 (95.4-100.0) | 50.0 (35.8-64.2) | 75.0 (65.6-83.0) | 100.0 (86.8-100.0) |
| 23h | 100.0 (95.4-100.0) | 42.3 (28.7-56.8) | 72.2 (62.8-80.4) | 100.0 (84.6-100.0) |
| 24h | 100.0 (95.4-100.0) | 30.8 (18.7-45.1) | 68.4 (59.1-76.8) | 100.0 (79.4-100.0) |

Values are percentages (95% CI), calculated using the Clopper-Pearson exact method. The time-to-positivity (TTP) threshold was varied while all other predictors were held constant. The 17-hour threshold (highlighted in yellow) is that of the original derivation. CI, confidence interval; NPV, negative predictive value; PPV, positive predictive value; TTP, time to positivity
