# Supplementary material for: External validation of a decision rule for bacteremia vs contaminants in pediatric blood cultures
Source: PLoS One. 2026 Nov 13;21(11):e0355010. doi: 10.1371/journal.pone.0355010 (PMC13766231; doi:10.1371/journal.pone.0355010)
Supplement: Supplementary file 6 [file pone.0355010.s006.docx]

**Re: Author response letter, Submission PONE-D-26-35518**	October 6, 2026

***External validation of a decision rule for bacteremia vs contaminants in pediatric blood cultures***

**Dear Academic Editor Iddya Karunasagar, dear *PLOS One* Editorial Team**,

We thank you for the careful evaluation of our above-mentioned manuscript and for the invitation to submit a revised version.

We are also most grateful to the reviewers for their positive comments and constructive criticism, which have allowed us to improve the clarity, methodological transparency, and interpretation of our work. We have revised the manuscript accordingly and provide a detailed, point-by-point response below. All changes have been incorporated in the revised manuscript and are shown in the marked-up version. Line numbers refer to that version.

**A) EDITORIAL REQUIREMENTS**

We have carefully reviewed each of the journal requirements in detail and have ensured that all applicable points have been fully addressed in the revised submission, which we believe is now aligned with the journal’s expectations.

**B) REVIEWER COMMENTS**

**REVIEWER 1**

**General assessment.** An interesting study with clinical relevance of blood stream infection in pediatric population.

**Response:** We thank the Reviewer for this positive assessment and for recognizing the relevance of the work to pediatric infectious disease.

**Comment 1, Section 1.2: Return visits of children with contaminated cultures**

**Comment:** *Specify symptomatic or asymptomatic children. If they are asymptomatic their return to the PED would be unlikely.*

**Response:** We thank the Reviewer for this helpful clarification. The return visit we refer to is not prompted by symptoms but by the laboratory notification of a preliminary positive result. Children who have already been discharged are actively recalled by the treating team for reassessment, frequently while asymptomatic or clinically improving. The Montreal cohort in which the rule was developed illustrates the magnitude of this phenomenon. Among the 104 children with a contaminant who had been discharged at the index visit, 40 (39%) were hospitalized and 37 (36%) were managed at a day center at follow-up, 70 (67%) received parenteral antibiotics, and only 27 (26%) were discharged home again (PMID: 38888530). We have rephrased the sentence accordingly (lines 66-73).

**Revised text:**

*“Children with contaminated cultures, who are often asymptomatic or clinically improving when the result becomes available, are frequently recalled to the PED by the treating team, undergo additional venipunctures, are admitted unnecessarily, and receive empirical antibiotics, with attendant risks of adverse events, line-related complications, and resistance selection [4, 12, 13, 16, 17]. In the Montreal cohort in which the rule was developed, two-thirds of children with a contaminant who had been discharged at the index visit received parenteral antibiotics after recall [11].”*

**Comment 2, Section 1.2: Suspected osteoarticular infection as a predictor**

**Comment:** *Please specify why so?*

**Response:** Thank you. Three complementary reasons explain why clinical suspicion of osteoarticular infection is a strong marker of true bacteremia, and we now state them briefly in the Introduction section (lines 93-100):

1) In children, acute osteomyelitis and septic arthritis are predominantly hematogenous infections and bacteremia is common. In the 2021 PIDS/IDSA guideline, blood cultures identified the causative organism in approximately one-third of children with acute hematogenous osteomyelitis (pooled positivity across 17 studies: 31.2%, 95% CI 26.3 to 36.0) (PMID: 34350458). A positive blood culture in a child with suspected osteoarticular infection therefore carries a high pretest probability of true bacteremia.
2) *Staphylococcus aureus*, the predominant pathogen, appears as Gram-positive cocci in clusters, the same morphology as coagulase-negative staphylococci, the most frequent contaminants. The Gram stain alone cannot separate them and clinical suspicion of osteoarticular infection supplies precisely the contextual information the Gram stain lacks and is therefore complementary to the Gram stain.
3) Suspected osteoarticular infection was the strongest independent predictor of true bacteremia in the Montreal cohort (adjusted odds ratio 43.6, 95% CI 16.2 to 118), present in 17-18% of bacteremias versus 3.1% of contaminants (PMID: 38888530). This explains its retention by the Classification and Regression Tree model of the original authors.

**Revised text:**

*“Several predictors discriminate true bacteremia from contaminants, including Gram-stain morphology, time to positivity (TTP), inflammatory markers, indwelling device, and suspected osteoarticular infection [11, 24-27]. The latter reflects the predominantly hematogenous pathogenesis of pediatric osteoarticular infections, in which blood cultures yield the causative organism, most often Staphylococcus aureus, indistinguishable from coagulase-negative staphylococci on Gram stain, in about one-third of cases [28]. It was the strongest independent predictor of true bacteremia in the Montreal cohort (adjusted odds ratio 43.6) [11].”*

**Comment 3, Section 1.2: Value of procalcitonin and C-reactive protein**

**Comment:** *Please explain why? Procalcitonin is a good indicator for bacterial infection of the blood stream.*

**Response:** We agree that procalcitonin is a valuable marker of invasive bacterial infection (IBI), and our original wording was too categorical. Procalcitonin is among the most accurate markers of IBI in young febrile infants (current references 29 and 30). In adults, it has shown promise for distinguishing contamination from true bacteremia due to coagulase-negative staphylococci (PMID: 34524647).

Our point concerns a narrower question. Once a culture is positive, the comparison group is not children without bacterial infection but children with contaminated cultures. These children were sampled precisely because they were febrile or unwell, and they frequently have a concomitant localized bacterial infection that raises inflammatory markers independently of bloodstream status. In the Montreal cohort, 43% of children with a contaminant had a bacterial diagnosis at the index visit, including pneumonia (13%) and urinary tract infection (9.4%) (PMID: 38888530). In addition, these markers are not measured systematically at the time of culture sampling. C-reactive protein was missing in 32-36% of the Montreal patients and could not be included in the derivation of the rule (PMID: 38888530 and PMID: 40810705). We have revised the sentence to acknowledge the value of procalcitonin while explaining this specific limitation (lines 101-108).

**Revised text:**

*“Procalcitonin and C-reactive protein are accurate markers of invasive bacterial infections in young febrile infants [29, 30]. Their value for this specific distinction in children with a positive blood culture is, however, less established. Children with contaminated cultures are themselves febrile or unwell and frequently have a concomitant localized bacterial infection that raises inflammatory markers independently of bloodstream status [11]. Moreover, these markers are not measured systematically at the time of culture sampling, which precluded the inclusion of C-reactive protein in the original derivation of the rule [24].”*

**Comment 4, Section 2.1: Consent and assent**

**Comment:** *Please specify consent and assent as the upper age limit was 16.*

**Response:** Thank you. Written informed consent for the further use of health-related data was available for all included participants and was obtained in two ways. First, when a signed institutional general consent of the Geneva University Hospitals was documented in the electronic health record, it was used. Second, for records without such a consent, all families were contacted by the research team, and written informed consent was obtained from the legal representative(s) for participants who were still minors at the time of contact (including those aged 14-17 years), or from the participants themselves when they had reached the age of majority. This procedure was approved by the Geneva Research Ethics Commission (CCER 2025-01069) under the Swiss Human Research Act, which governs the further use of health-related personal data. Because the study consisted solely of the retrospective review of existing records, without any intervention on participants, no separate assent procedure was required by the ethics committee. We have now specified this in the revised Methods section (lines 133-141) and the inclusion criterion in Section 2.2. has been harmonized accordingly (lines 147-151).

**Revised text (Section 2.1):**

*“Written informed consent for further use of health-related data was obtained for all participants, in accordance with the Swiss Human Research Act [34]. When available, the institutional general consent of the Geneva University Hospitals, documented in the electronic health record, was used. Otherwise, families were contacted and written informed consent was obtained from the legal representative(s) for participants who were still minors at the time of contact, or from the participants themselves if they had reached the age of majority. As the study involved only retrospective review of existing records, no separate assent procedure was required by the ethics committee.”*

**Revised text (Section 2.2, inclusion criterion):**

*“… and written informed consent for the further use of health-related data, either through the institutional general consent or from the legal representative(s), or from the participant if they had reached the age of majority at the time of contact.”*

**Comment 5, Section 2.2: Retrospective or prospective design**

**Comment:** *Was this a retrospective or prospective study? The abstract mentions this as a retrospective study.*

**Response:** The study was entirely retrospective. We apologize for the confusing wording. “Random ordering” refers to the order in which existing records were screened. All data were abstracted retrospectively from the microbiology database and the electronic health record, and no patient was enrolled prospectively. We have now revised the Participants section 2.2 (lines 158-164) and, to avoid any ambiguity, we have replaced “enrollment/enrolled” with “inclusion/included” throughout the manuscript and in Figure 2.

**Revised text:**

*“To minimize selection bias, eligible records were randomly ordered before retrospective screening, and inclusion continued until the prespecified sample size was reached. Because inclusion was capped at the target sample size, positive cultures not screened by that point were not assessed. Included patients therefore represented a random subset of all eligible cases rather than a clinically selected sample. All data were abstracted retrospectively from existing records. No patient was enrolled prospectively.”*

**Comment 6, Section 2.4: Timing of the initial Gram stain**

**Comment:** *Specify 'initial Gram stain. When was this done?*

**Response:** The initial Gram stain is the one performed by the bacteriology laboratory directly from the blood culture bottle once the BD BACTEC FX system has flagged microbial growth. It is the first microbiological result communicated to the treating physician (preliminary positive report), before organism identification, and it is the moment at which the CDR is intended to be applied (PMID: 40810705). When the system flagged a bottle outside laboratory operating hours, the Gram stain was performed the next morning upon reopening of the laboratory. This delay affects when the results is communicated to the clinician, but not the measured TTP, which ends at the instrument alarm. We have clarified this in the Methods section (lines 193-195).

**Revised text:**

*“Gram-negative organisms OR Gram-positive cocci arranged in pairs or chains on initial Gram stain, performed on the positive bottle before organism identification (the next morning for bottles flagged after laboratory hours) (high-risk gate).”*

**Comment 7, Section 2.7: Reference standard**

**Comment:** *Specify the reference standard*

**Response:** We agree. The reference standard is fully described in Section 2.3. We now name it explicitly in the Statistical Analysis and cross-refer that section (lines 235-238).

**Revised text:**

*“The four CDR predictors were applied to each case, and the resulting risk classification (high or moderate risk, CDR-positive; low risk, CDR-negative) was compared with the reference standard (adjudicated true bacteremia versus contaminant; see section 2.3).”*

**Comment 8, Section 4.2: Effect of delayed loading on TTP**

**Comment:** *The sentence seems contradictory. Delayed loading would lead to delayed TTP.*

**Response:** We thank the reviewer for pointing out this ambiguity, which arises from two different time intervals. In our study, TTP was defined as the time from placement of the bottle into the BD BACTEC FX system to the detection of growth (Section 2.4). When a bottle is held at room temperature before loading, microorganisms continue to multiply during this pre-incubation period. Once the bottle is loaded, the inoculum is therefore larger and the instrument flags growth sooner, so that the instrument-measured TTP is shortened. Conversely, the total time from blood collection to detection is prolonged, because the pre-incubation delay is added. This pattern has been documented for BACTEC systems (PMID: 41763125), in which “*Higher preincubation times were partially mitigated by a lower incubation time, with an overall increase in total detection time*” (PMID: 35079040), and for other continuous-monitoring systems (PMID: 33034779). We have rewritten the sentence to make this distinction explicit (lines 382-385)

**Revised text:**

*“The TTP predictor depends on pre-analytical conditions. When bottles are held at room temperature before loading, microbial growth begins before incubation, so that the instrument-measured TTP, from loading to detection as defined in this study, is shortened, whereas the total time from blood collection to detection is prolonged [33, 43].”*

**Comment 9, Section 4.5: Discrimination by procalcitonin and C-reactive protein in our cohort**

**Comment:** *However, your study did show discrimination between true infection and contaminant with Procal, and CRP. Please explain.*

**Response:** We agree and thank the reviewer. Our wording was too categorical. Both markers were markedly higher in true bacteremia than in contaminants (Table 1). While re-examining these data, we identified and corrected a transcription error in the procalcitonin medians and interquartile ranges reported in Table 1. The between-group difference and P value were unaffected. This is consistent with the Montreal data (median C-reactive protein 55 versus 14; PMID: 38888530) and with pediatric evidence for procalcitonin (PMID: 31097470). However, these group-level differences do not establish the individual-level discrimination required to safely withhold antibiotics in a given child. For both markers, the interquartile ranges overlapped. Moreover, procalcitonin was available in only 92 of 130 children (71%; 56/78 true bacteremia and 36/52 contaminants). Importantly, it was measured at the clinician's discretion rather than systematically, and may therefore reflect selective testing in more severely ill children. Neither marker is part of the evaluated rule, and both were recorded as descriptive variables only (Section 2.3). We have rewritten the paragraph to report this finding explicitly and to frame the incremental value of procalcitonin as a question for prospective evaluation with systematic measurement (lines 433–450).

**Revised text:**

*“Our findings also qualify the role of biomarkers. In our study population, C-reactive protein and procalcitonin were significantly higher in true bacteremia. However, group-level differences do not establish the individual-level discrimination required to safely defer antibiotics. Their distributions overlapped, and procalcitonin was measured in only 71% of children, at the clinician’s discretion. Moreover, neither marker, nor composite tools such as the Lab-score were designed for the binary task of distinguishing true bacteremia from contamination. They were validated in broader febrile populations rather than in children with a preliminary positive blood culture [29, 30, 52]. Structured rules combining microbiological, clinical and host predictors therefore remain more likely than biomarkers alone to support management changes in this scenario. Whether procalcitonin adds incremental value to the CDR deserves prospective evaluation with systematic measurement at the time of culture sampling.”*

**Comment 10, Section 4.7: Limitations**

**Comment:** *Clinical correlation and positivity.*

**Response:** We thank the reviewer. We understood this comment as a request to acknowledge two features of the rule that are relevant to its interpretation. First, the rule does not incorporate clinical correlation at the time of the result, such as the child’s clinical appearance or the persistence of fever. Second, it relies on a single positive culture and does not use the number of positive blood-culture sets, which is a classic criterion for distinguishing true bacteremia from contamination, as surveillance definitions require identification in two or more blood specimens collected on separate occasions (Chapter 4, in 2026 National Healthcare Safety Network (NHSN) Patient Safety Component Manual, available here: <https://www.cdc.gov/nhsn/pdfs/pscmanual/pscmanual_current.pdf>). This information is rarely available in pediatric practice, where a single culture is usually drawn. We have added this as a limitation (lines 485-487). Should the reviewer have intended a different point, we would be glad to address it further.

**Revised text:**

*“Sixth, the CDR relies on a single positive culture and does not incorporate the child’s clinical status at the time of the result, nor the number of positive culture sets [55]. It should therefore support, not replace, clinical reassessment.”*

**Comment 11, Figure 2**

**Comment:** *Please comment on this discrepancy.*

**Response:** The 16 CDR-positive contaminants are not a data inconsistency but the false positives of the rule (10 classified at high risk and 6 at moderate risk), which correspond to the observed specificity of 69.2% (36/52). This is the expected consequence of a rule optimized for sensitivity. In its derivation, the Classification and Regression Tree model penalized a false negative 40 times more heavily than a false positive (PMID: 40810705). Table 1 shows where these false positives come from. The 10 high-risk false positives correspond exactly to the contaminants with a high-risk Gram morphology (6 Gram+ cocci in chains and 4 Gram- bacilli). The 6 moderate-risk false positives were triggered by a time to positivity below 17 hours in at least 5 cases, since only 1 contaminant occurred in a child with an indwelling device and none in a child with suspected osteoarticular infection.

To make Figure 2 self-explanatory, the screening flow now reports the number of cultures at each step, and each outcome box is labelled as true/false positives and negatives. And “enrolled” was replaced with “included”.

**REVIEWER 2**

**General assessment.** The article is scientifically sound and well written. Some minor queries and comments.

**Response:** We are grateful for the reviewer’s positive assessment.

**Comment 1: Restriction of the clinical criterion to osteoarticular infection**

**Comment:** *Why is only suspicion of osteoarticular infection considered? Many a times information about infections like UTI and pneumonia also give a major clue on true bacteremia.*

**Response:** We thank the reviewer for this thoughtful comment. As an external validation study, our objective was to test the Sainte-Justine rule exactly as derived and published (PMID: 40810705). Adding or modifying predictors would amount to updating the model, which would require its own derivation and validation and lies beyond the scope of this work. Nevertheless, the original data and the literature provide a clear rationale for why osteoarticular infection, but not urinary tract infection and pneumonia, was retained:

First, urinary tract infection and pneumonia did not discriminate in the source data. In the Montreal cohort (PMID: 38888530), urinary tract infection was equally frequent among true bacteremia (9.8%) and contaminants (9.4%). And pneumonia was more frequent among contaminants (3.1% vs 13%). A bacterial infection treated with antibiotics at the index visit was only weakly associated with true bacteremia (adjusted odds ratio 2.03, 95% CI 1.17 to 3.53), whereas suspected osteoarticular infection was the strongest independent predictor (adjusted odds ratio 43.6, 95% CI 16.2 to 118). Of the 18 candidate predictors defined from a literature review and expert consensus, the final model retained only the four most discriminant ones (PMID: 38888530, PMID: 40810705).

Second, bacteremia accompanies these infections at very different rates. Pediatric acute osteomyelitis and septic arthritis are predominantly hematogenous and blood cultures identify the causative organism in about one-third of cases (pooled positivity across 17 studies: 31.2%, 95% CI 26.3 to 36.0) (PMID: 34350458). A positive blood culture in a child with suspected osteoarticular infection therefore carries a high pretest probability of true bacteremia. By contrast, bacteremia occurs in 6.5% of febrile infants with urinary tract infection (PMID: 21098155) and in 2.5% of children hospitalized with community-acquired pneumonia who had a blood culture obtained (PMID: 28835382). A diagnosis of UTI or pneumonia therefore frequently coexists with a contaminated blood culture.

Third, osteoarticular infection adds information that the Gram stain cannot provide. The usual bloodstream pathogens of UTI (Gram- bacilli such as *E. coli*) and pneumonia (Gram+ cocci in pairs such as *S. pneumoniae*) are already captured by the high-risk Gram gate. In contrast, *S. aureus*, the predominant pathogen of osteoarticular infections, appears as Gram+ cocci in clusters indistinguishable from coagulase-negative staphylococci, the most frequent contaminants. Hence, clinical suspicion of osteoarticular infection supplies precisely the context that the Gram stain lacks.

We have summarized this rationale in the Introduction section (lines 95-100) and added a sentence to the Discussion section (lines 452-458).

**Revised text:**

*“Clinical foci other than osteoarticular infection, such as urinary tract infection or pneumonia, are not part of the CDR. In the derivation data, these diagnoses did not discriminate true bacteremia from contamination [11], likely because bacteremia complicates only a minority of these common infections [53, 54] and their usual bloodstream pathogens are already captured by the Gram-stain high-risk gate. Whether incorporating such foci could further improve the rule should be explored in future model-updating studies.”*

We thank both reviewers and believe the manuscript is substantially clearer and more measured as a result of their input.

Johan N. Siebert, MD
